# Extensive RNA editing and splicing increase immune self-representation diversity in medullary thymic epithelial cells

**DOI:** 10.1186/s13059-016-1079-9

**Published:** 2016-10-24

**Authors:** Miri Danan-Gotthold, Clotilde Guyon, Matthieu Giraud, Erez Y. Levanon, Jakub Abramson

**Affiliations:** 1 The Mina and Everard Goodman Faculty of Life Sciences, Bar-Ilan University, Ramat-Gan, 52900 Israel; 2 Department of Infection Immunity and Inflammation, Cochin Institute, Paris, France; 3 Department of Immunology, Weizmann Institute of Science, Rehovot, Israel

**Keywords:** Medullary thymic epithelial cells (mTECs), Thymus, Alternative splicing, RNA editing, RNA sequencing, Self-tolerance

## Abstract

**Background:**

In order to become functionally competent but harmless mediators of the immune system, T cells undergo a strict educational program in the thymus, where they learn to discriminate between self and non-self. This educational program is, to a large extent, mediated by medullary thymic epithelial cells that have a unique capacity to express, and subsequently present, a large fraction of body antigens. While the scope of promiscuously expressed genes by medullary thymic epithelial cells is well-established, relatively little is known about the expression of variants that are generated by co-transcriptional and post-transcriptional processes.

**Results:**

Our study reveals that in comparison to other cell types, medullary thymic epithelial cells display significantly higher levels of alternative splicing, as well as A-to-I and C-to-U RNA editing, which thereby further expand the diversity of their self-antigen repertoire. Interestingly, Aire, the key mediator of promiscuous gene expression in these cells, plays a limited role in the regulation of these transcriptional processes.

**Conclusions:**

Our results highlight RNA processing as another layer by which the immune system assures a comprehensive self-representation in the thymus which is required for the establishment of self-tolerance and prevention of autoimmunity.

**Electronic supplementary material:**

The online version of this article (doi:10.1186/s13059-016-1079-9) contains supplementary material, which is available to authorized users.

## Background

Central tolerance is established in the thymus, where immature T lymphocytes are instructed by thymic stroma to become immunocompetent cells capable of recognizing foreign invaders while tolerating the body’s own components. This process is primarily mediated by both negative selection of potentially self-reactive T cell clones and the induction of CD25^+^, Foxp3^+^ T regulatory (T_reg_) cells by various thymus-resident antigen-presenting cells [1]. In particular, medullary thymic epithelial cells (mTECs) were shown to play the main role in this process, because of their unique ability to promiscuously express and subsequently present a large spectrum of the body’s self-antigens, including those that normally have high tissue restriction [2]. The expression of transcripts encoding many of these tissue-restricted antigens (TRAs) is regulated by a single transcriptional regulator: the autoimmune regulator Aire [3]. Indeed, mice with a dysfunctional *Aire* gene express only a fraction of the TRA repertoire and as a result develop autoantibodies and immune infiltrates directed at multiple peripheral tissues [3]. Similarly, human patients with dysfunctional *AIRE* gene suffer from a devastating Autoimmune polyendocrine syndrome type 1 (APS1) [4, 5].

RNA-processing mechanisms, in particular alternative splicing (AS) and RNA editing, enable the production of multiple mRNA transcripts from the same gene, thereby expanding the diversity and complexity of individual gene products. Consequently, a single gene may give rise to different protein variants with different functional roles in different tissues [6, 7]. Current estimates suggest that ~95 % of genes with more than one exon undergo AS [8, 9] and in extreme cases one gene can give rise to thousands of different isoforms [10]. In many cases, AS preserves the protein open reading frame, leading to the expression of different protein isoforms, which frequently have different functional properties [11, 12]. A common consequence of AS in metazoans is insertion or deletion of entire segments of a protein as a result of an in-frame cassette exon insertion or exclusion [13].

Transcript and protein diversities are further increased by various RNA-editing mechanisms, which induce single nucleotide substitution in the RNA, which may thereby alter the protein sequence and function. In mammals, RNA editing involves mainly deamination of adenosine (A) to inosine (I) (recognized as guanosine (G) by all molecular machineries) mediated by the adenosine deaminases acting on RNA (ADARs) protein family [14, 15]. These enzymes have a large number of targets in both human and mice and frequently edit several sites in clusters and may lead to changes in several amino acids in a single protein [16–20]. It is also noteworthy that ADAR activity was first isolated from a calf thymus [21]. Another, less prevalent, type of RNA editing involves deamination of cytosine (C) to uridine (U) by the APOBEC protein family of cytidine deaminases, mainly Apobec-1 [22, 23]. Until recently, the only gene reported to undergo codon alteration caused by C-to-U editing was Apolipoprotein B (ApoB) [24, 25]. Interestingly, ApoB is an Aire-dependent tissue-restricted antigen, whose expression is mainly restricted to the small intestine and liver. More recently, additional transcripts were shown to undergo C-to-U editing in their coding sites by Apobec3A in humans [26], or by Apobec1 within their 3' UTRs in mice [27, 28].

RNA processing in the peripheral tissues may increase the repertoire of potentially immunogenic epitopes, which may then be recognized as non-self-peptides by the adaptive immune system. This is particularly true for any protein variant, which was not presented to the developing T cells in the thymus [29]. For instance, a splice variant of mouse PLP gene was shown to be expressed in oligodendrocites, but not in the thymus, suggesting that absence of the specific exon in the thymus may result in loss of tolerance to the relevant polypeptide in the periphery [30].

Here, we evaluate the extent of various co-transcriptional and/or post-transcriptional RNA process products expressed in mTECs, assess the intrinsic diversity of the individual self-antigen transcripts, and compare it with that of other cell types and tissues. To this end, we analyzed available RNA-sequencing (RNA-seq) datasets and determined the extent and diversity of gene expression, AS and RNA editing in mTECs in comparison with other tissues and cell types in the body. Indeed, our analyses demonstrate that, on average, mTECs express 3000–6000 more genes than other tissues. Interestingly, the extent of representation of the individual tissues within mTECs is very diverse, ranging from relatively low (e.g. testis or brain) to high (e.g. colon or skin) levels of tissue-specific coverage. Moreover, our results reveal that mTECs display a relatively high level of AS and RNA editing, which thus helps to further expand the already broad repertoire of self-antigens in the thymus. Our results therefore highlight yet another level by which the immune system assures a comprehensive representation of the body’s own proteins in the thymus.

## Results

### Medullary thymic epithelial cells express ~85 % of the entire coding genome

Although promiscuous gene expression in mTECs is a well-established biological phenomenon [31], the extent of such expression, especially in comparison with other tissues, has not yet been analyzed in a comprehensive manner using state-of-the-art methodologies. Therefore, to better determine the fraction of genes expressed in mTECs and other mouse tissues, we took advantage of RNA-seq technology. Specifically, we performed a comparative analysis of RNA-seq datasets obtained from different mTECs populations as well as ten different tissues and epithelial cell populations, including brain, testes, liver, kidney, lung, colon, skeletal muscle (skm), spleen, cortical thymic epithelial cells (cTECs), and skin epithelial cells (skinEC) [32, 33]. The mTEC populations included: (1) MHC-II low mTECs (mTEC^lo^), which mainly represent an immature mTEC population; (2) MHC-II high mTECs (mTEC^hi^), mainly representing a mature population; and (3) an Aire-deficient mTEC^hi^ population (AireKO), which is severely impaired in promiscuous gene expression (see “Methods”).

**Fig. 1:**
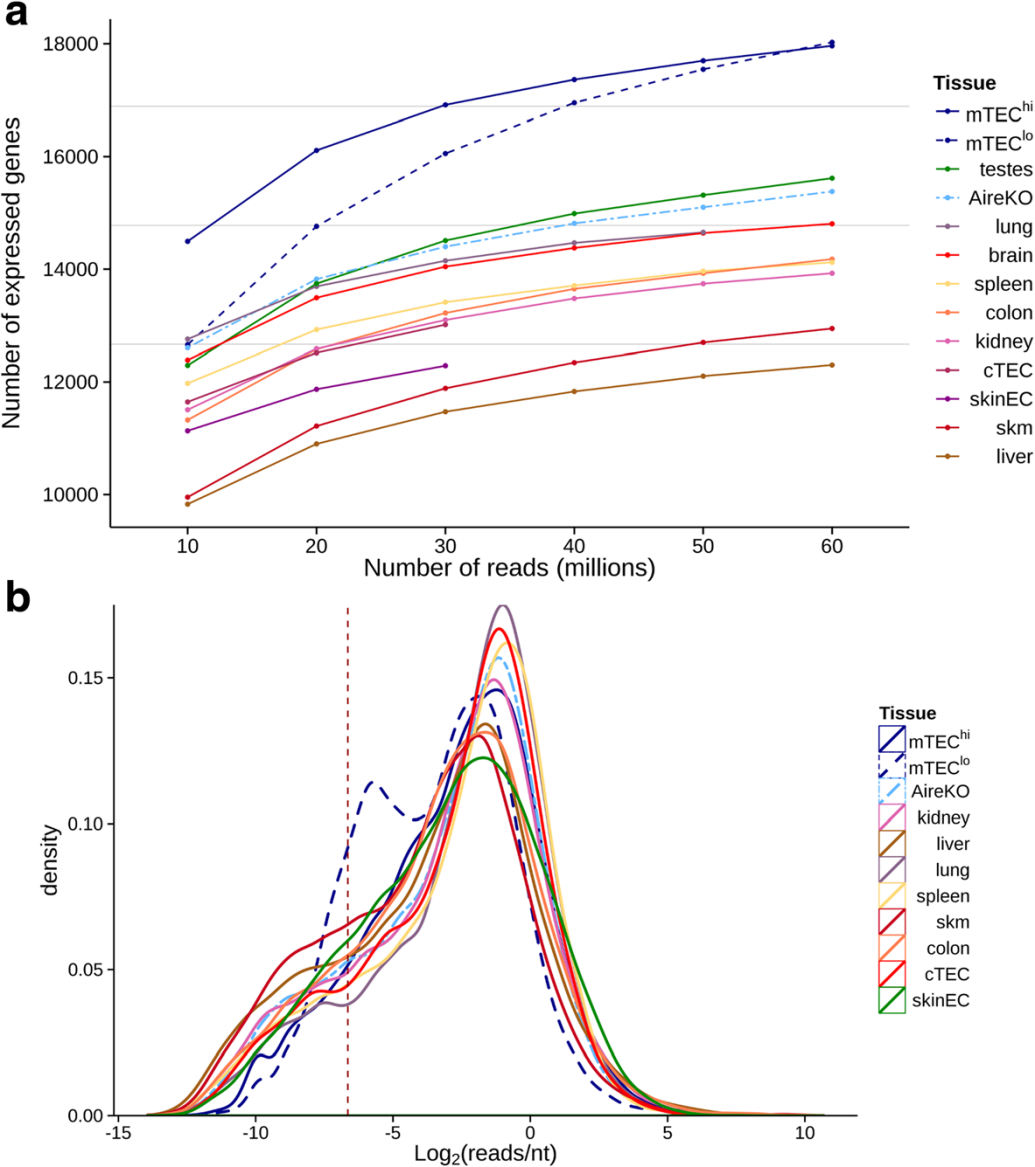
mTECs express higher number of genes compare to other tissues. **a** Mature mTECs expressed higher number of genes than all the tissues and cell types examined, including the brain and testes. Number of protein-coding genes expressed (at least 0.01 reads/nt) in eight different tissue types (brain, testes, colon, kidney, liver, lung, skeletal muscle (skm), and spleen), two epithelial cell types: cortical thymic epithelial cells (cTECs) and skin epithelial cells (skinEC), and three types of mTECs (low MHC-II [mTEC^lo^], high MHC-II [mTEC^hi^], and Aire-deficient [AireKO]) on the basis of 10–60 million randomly selected aligned RNA-seq reads for each sample. *Gray lines* denote the percentage of genes out of 21,111 accounted in this analysis. **b** Distribution of gene expression levels of immature mTECs exhibit a high ratio of lowly expressed genes and is different from other tissues and cell types examined. Kernel density estimates of normalized read counts distributions of 50 million randomly selected aligned reads from the same RNA-seq data within protein-coding genes. *Vertical line* denotes the expression detection threshold used in Fig. 1a (0.01 reads/nt)

The analysis, using the selected cutoff (see “Methods”), revealed that most of the tissues express 12,000–14,000 genes (i.e. 60–65 % of the coding genome) (Fig. 1a). The lung and two immunologically privileged sites, brain and testis, were the only tissues that expressed a larger fraction of the genome, in the range of 70–75 %. This was not entirely surprising, as enhanced global gene expression in the brain and testis has been reported in the past by several independent studies [34–37]. In line with other recently published studies, the mTEC^hi^ population expressed nearly 18,000 genes, which represents ~85 % of the coding genome, while Aire-deficient mTECs (AireKO) expressed approximately 15,000 genes, suggesting that Aire is responsible for the induction of ~3000 genes in mTECs [38–40]. Interestingly, even in the absence of Aire, the mTECs expressed a relatively large fraction of the genome (~75 %), considerably exceeding the overall genome expression in other peripheral tissues (Fig. 1a). Interestingly, neither cTECs nor skinEC demonstrated higher overall genome expression than other tissues, suggesting that promiscuous gene expression is indeed unique to the mTECs population (Fig. 1). Notably, the mTEC^lo^ population was also characterized by a strong promiscuous gene expression. However, the levels of many individual transcripts were dramatically lower than in the mTEC^hi^ population as clearly illustrated in Fig. 1b.

### Differential projection of tissues’ self-shadows in mTECs

Although mTECs are characterized by a very high level of promiscuous expression of TRA genes (Fig. 1), whether mTECs mirror all peripheral tissues to the same extent has not yet been thoroughly analyzed. To better address this question, we analyzed the level of overlap of tissue-restricted genes for each individual tissue (or cell type) by performing leave-one-out analysis and TRA detection on all RNA-seq datasets. For this analysis, a TRA gene was defined as a gene that was highly expressed (Fragments Per Kilobase of transcript per Million mapped reads (FPKM) > 5) in one of the tissues examined and was either lowly (FPKM < 0.3) or not expressed in the other analyzed tissues (except mTECs).

**Fig. 2:**
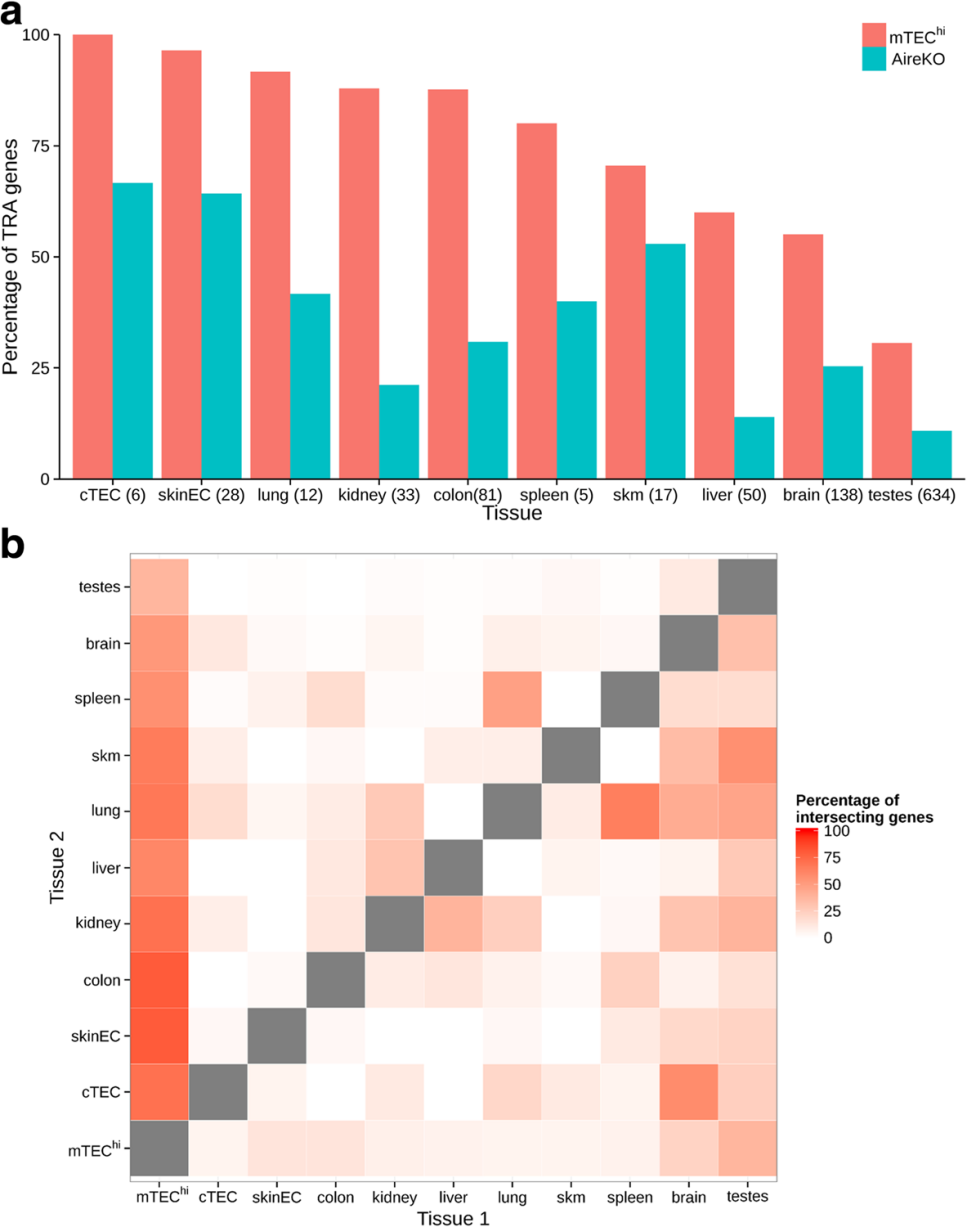
mTEC cells express higher percentage of tissue specific genes compare to any other tissue. **a** *Percentage* of the tissue restricted antigens (TRA) genes of each tissue expressed in mature mTECs (mTEC^hi^) and Aire-deficient (AireKO) cells. The number of TRA genes expressed in the tissue is indicated in *parentheses*. A relatively low fraction of TRA genes of testes and brain are expressed in mTECs. **b** *Leave-one-out analysis* comparing TRA expression across all tissues. The percentage of the coding genes uniquely expressed in both tissue 1 and tissue 2 (and not in other tissue examined) out of the genes expressed only in tissue 1 (and not in other tissue except tissue 2) are presented. The ratio of expressed TRA is the highest in mTECs compare to other tissues. *cTEC* cortical thymic epithelial cells, *skinEC* skin epithelial cells, *skm* skeletal muscle

As expected, most of the analyzed peripheral tissues were, to a large extent, mirrored in the mTEC^hi^ population (Fig. 2a), which expressed ~60–100 % of their tissue-specific gene signature. In contrast, the brain and testes demonstrated a relatively small overlap of their specific TRA gene signatures with the mTEC^hi^ population. Specifically, mTECs expressed only 31 % of testis-specific and 55 % of brain-specific antigens (Fig. 2a), indicating that many genes specific to these two immunologically privileged tissues are not mirrored on the “central level” by mTECs.

The overlap between mTECs and other tissues dramatically decreased to 14–67 % in the Aire-deficient mTEC^hi^ population (Fig. 2a). Importantly, leave-one-out analysis with different parameters (see “Methods”), in which we removed one sample for the TRA detection step and then detected the ratio of TRAs of each of the other samples expressed in the removed sample, further supported these results and also demonstrated that other tissues have dramatically lower TRA overlap between themselves (3–48 %), with a mean percentage lower than 15 % (Fig. 2b and Additional file 1: Figure S1).

### mTECs are characterized by a high number of AS events per gene

As discussed above, AS of a single gene can give rise to several different protein variants, which differ in their amino acid sequences [10]. This phenomenon further increases the diversity of self-antigens in specific tissues and/or different developmental stages and creates an additional challenge for the immune system to avoid self-reactivity [10, 41]. Therefore, to better assess the degree of AS in mTECs and in other cell types and tissues, we counted the number of splice junctions detected for each coding gene in the RNA-seq datasets that were available to us. In order to have a comparable and uniform set of reads from each of the various samples, we randomly selected 50 million reads from each sample and trimmed long reads to 80 nucleotides.

**Fig. 3:**
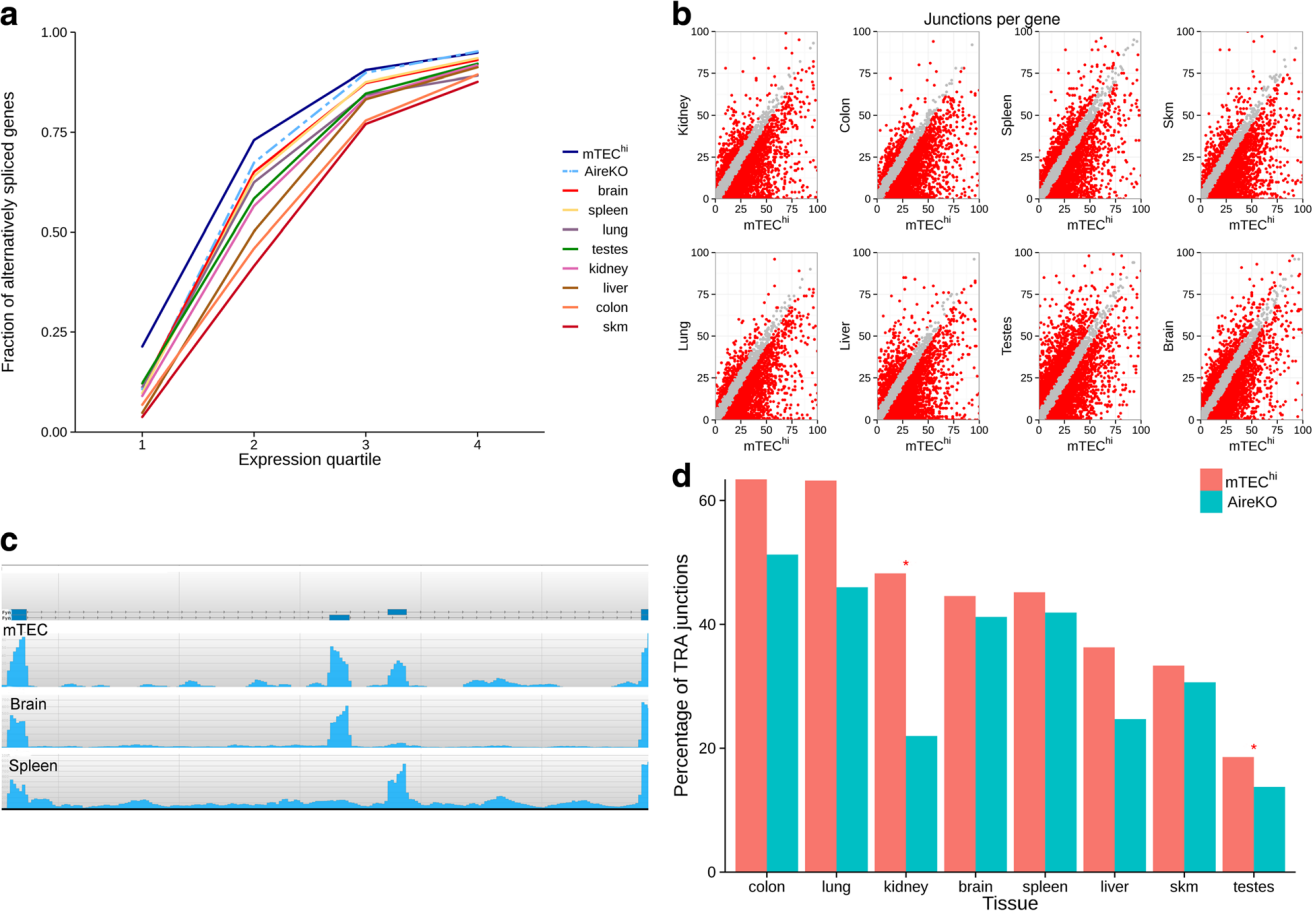
High level of AS in mTECs compared to other tissues. **a** Mature mTECs express the highest rate of alternatively spliced genes. A fraction of alternatively spliced genes detected in each tissue in four levels of gene expression (by quartiles). **b** Mature mTECs express a higher number of splice junctions per gene compare to other tissues. *Dot plot* of splice junctions number detected for each gene expressed in both mature mTECs (mTEC^hi^) and each sample examined. *Red dots* denote a difference of more than five splice junctions per gene, *gray dots* denote five and fewer splice junctions difference per gene. *Axes* were limited between 0–100 splice junctions per gene. **c** Fyn alternative splicing pattern in mTECs, brain and spleen. Reads coverage of mutually exclusive exons in each tissue is presented. **d** Similar rate of tissue restricted spliced junctions is expressed in mature mTECs and AireKO samples. Fraction of tissue restricted spliced junctions (TRA junctions) expressed in mature mTECs and AireKO sample. *Asterisk* denotes a statistically significant change between mTEC^hi^ and AireKO sample (*p* value ≤ 0.05)

We first compared the fraction of alternatively spliced genes out of all expressed coding genes that have multiple exons in each sample. We excluded mTEC^lo^ cells from this type of analysis due to their high ratio of lowly expressed genes and abnormal gene expression pattern (Fig. 1b), which could therefore influence the results for this analysis. Our results clearly demonstrate that from all analyzed cell types and tissues, mature mTECs express the highest fraction of alternatively spliced protein-coding genes, independently of their expression levels (Fig. 3a). The higher ratio of alternatively spliced genes in the lower quintile group may indicate that the concentration of each splice variant in these cells is relatively uniform. This is in line with previously reported data demonstrating that mTECs display high splicing entropy [42].

Next, we examined the number of splice junctions detected in each gene in mTECs versus other tissues. For most of the genes, mTECs expressed either equal or higher numbers of splice junctions (Fig. 3b). This was also observed in the testes and brain, which were previously shown to have complex AS patterns [34, 43]. Similar results were obtained when analyzing only moderately expressed genes (FPKM < 2, Additional file 1: Figure S2), demonstrating that the complex splicing pattern is not a result of high expression level of these genes in mTECs. Moreover, Multivariate Analysis of Transcript Splicing (rMATS) [44] also demonstrated that mTECs express more splice variants compared to other tissues except for the brain (Additional file 1: Table S2). A good example is the Src tyrosine kinase Fyn, which is highly expressed in the hematopoietic system and in the brain. As can be seen in Fig. 3c, it exists in two major alternatively spliced forms, which are either hematopoietic-specific or brain-specific [45]. Importantly, both alternative forms were found in mTECs in relatively equal levels, suggesting that mTECs cover various tissue-specific splice variants of the same gene in relatively equal distribution.

In addition, we also examined the level of tissue-restricted splice junctions, which are uniquely found only in one, but not in the other tissues, whereas the gene was expressed in other tissues. The rate of tissue-restricted splice junctions expressed in mTECs differed from tissue to tissue, with relatively high rates for the colon or lung to lower relative rates for the skin or skeletal muscle (Fig. 3d). Overall, the fraction of tissue-restricted splice junctions covered by mTECs was lower than the whole gene TRA coverage for all tissues, indicating splicing isoforms representation by mTECs is less comprehensive. These results were consistent also when using different number of randomly selected reads from the mTEC^hi^ sample (Additional file 1: Figure S3). Moreover, we also examined the levels of tissue-restricted splice junctions with pre-known AS events that are annotated in the UCSC database (Alt Events track) [46]. Indeed, this analysis further demonstrated that mTECs express high rates of tissue-restricted splice junctions of most of the tissues examined (Additional file 1: Figure S4).

Surprisingly, in contrast to the previously published study [42], the extent of representation of splice isoforms was comparable between the wild type (WT) and the Aire-deficient mTECs for most of the tissue-specific transcripts (Fig. 3d), suggesting that the high level of AS in mTECs is, to a large extent, Aire-independent. The only exceptions were kidney-specific and testis-specific transcripts, which showed a reduced rate of splice junctions in Aire-deficient mTECs (Fig. 3d). Similar results were obtained when only genes with high expression level were included in the analysis (FPKM > 2, Additional file 1: Figure S5).

### mTEC cells are characterized by high RNA-editing rate

**Fig. 4:**
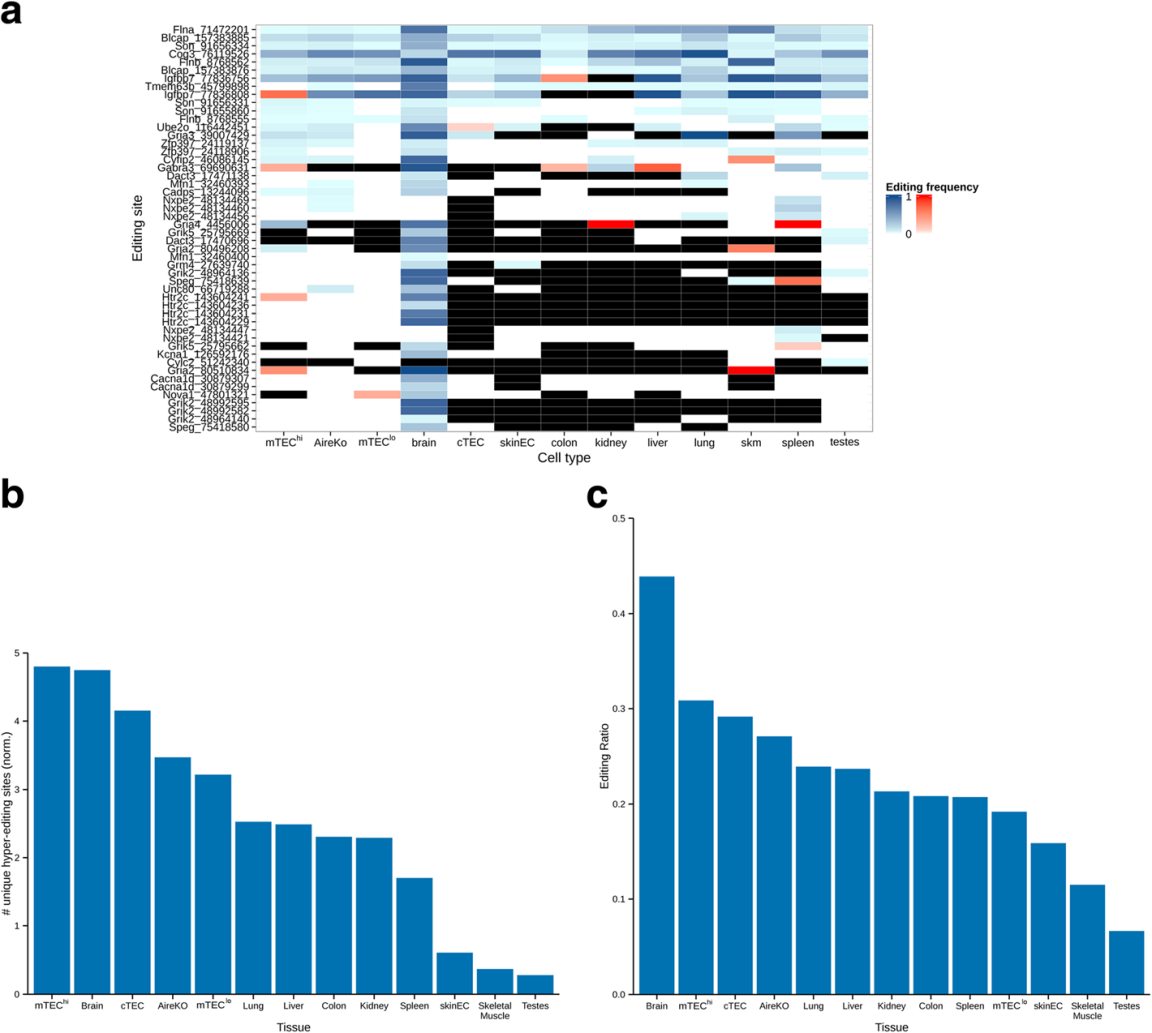
Higher rate of RNA-editing sites were detected in mTEC cells (A to I). **a** *Heatmap* of RNA-editing frequency in known non-synonymous RNA-editing sites (A to I) edited in at least one of the examined cell types or tissues. *Blue rectangle* denotes editing site supported by ten reads or more (edited and non-edited reads), *red rectangle* denotes editing site supported by less than ten reads, *white rectangle* denotes no edited reads were found, *black rectangle* denotes no reads were found in this editing site. The highest editing level in these non-synonymous sites was found in the brain. Mature mTECs and AireKO samples have relatively similar editing levels in these sites. **b** The number of hyper-editing sites in mTECs is comparable to the brain. The number of unique hyper-editing sites (A to I) per million aligned reads detected in each sample. **c** Global RNA-editing rate in mature mTECs is second to the brain and very similar to cTECs. The ratio of edited sites out of all editable sites expressed in each sample is represented

In addition to AS, protein diversity is further expanded by RNA editing, which can make discrete changes to specific nucleotide sequences within an RNA molecule after it has been generated by RNA polymerase [47]. Two types of RNA mammalian nucleobase modifications are known, adenosine (A) to inosine (I) and cytidine (C) to uridine (U) deaminations. These editing reactions are mediated by two RNA-editing families of enzymes, Adar and Apobec, respectively [14, 48]. Although most A-to-I RNA-editing events are taking place within non-coding regions of the genome, editing in coding genes may cause a non-synonymous change at the protein level. In order to assess the extent and distribution of A-to-I RNA editing with an effect at the proteome level, we examined the editing frequency of all editing sites in the RADAR database [18] in addition to sites obtained from the hyper-editing analysis (see below) with a non-synonymous effect on the derived protein (i.e. 50 unique sites in total, edited (supported by at least five reads) in one or more of the tissues examined) (Fig. 4a). Out of 29 sites covered by at least ten reads in mTEC^hi^ sample, 19 sites (i.e. 65.5 %) were found to undergo RNA editing. In the vast majority of these recoding edited sites in mTECs (73.7 %), RNA-editing levels were lower than 10 %, suggesting that RNA editing in mTECs may follow a similar stochastic pattern as promiscuous gene expression. Moreover, these data imply that accurate assessment of the rate of editing in these sites may require a higher coverage.

In order to overcome this limitation, we further explored the global rate of A-to-I RNA editing in mTECs in comparison to other tissues. For this, we counted the number of hyper-edited sites in each tissue sample, using a newly devised pipeline which detects heavily edited reads [16]. While the normalized number of unique hyper-edited sites in typical tissues was <3 unique editing sites per million aligned reads (Fig. 4b), mTEC^hi^ cells demonstrated 4.80 such sites per million aligned reads. This number is comparable to the number observed in the brain (4.75) (Fig. 4b), which has been reported to have the highest RNA-editing capacity from all different tissues [49]. Notably, cTECs also had a high number of unique hyper-editing sites but less than the number that was detected in mTECs (4.15).

Since the number of hyper-edited sites may be affected by the number of genes expressed in the sample, we also examined the editing ratio within each sample (i.e. the ratio of editable sites versus edited sites, see “Methods”). Similar to the above data, the brain showed the highest ratio of edited sites (0.44) (Fig. 4c), which was followed by the mature mTEC (0.31), cTEC (0.29), and Aire-deficient mTECs (0.27). As in the hyper-editing analysis, the results obtained for the cTEC sample were similar to mTECs indicating that the process responsible for the higher ratio of edited sites in mTECs may occur also in cTECs. These high RNA-editing levels are probably associated with *Adar1* since its expression levels in mTEC^hi^ and cTEC samples were comparable to the level in the brain tissue, whereas *Adar2* expression levels were significantly higher in the brain (Additional file 1: Table S3).

In addition, we have performed an additional analysis in which we examined RNA-editing levels only in sites that are significantly different between mTECs and the other individual tissue. In order to overcome biases derived from the different number of reads in the samples, we used comparable number of reads in each of the samples (30 M randomly selected reads). Indeed, the vast majority of statistically significant altered events had a higher A-to-I editing rate in mTECs vis-à-vis other tissues, except for mTEC^lo^ (Fisher’s exact test, false discovery rate (FDR) ≤ 0.05) (Additional file 1: Table S4).

**Fig. 5:**
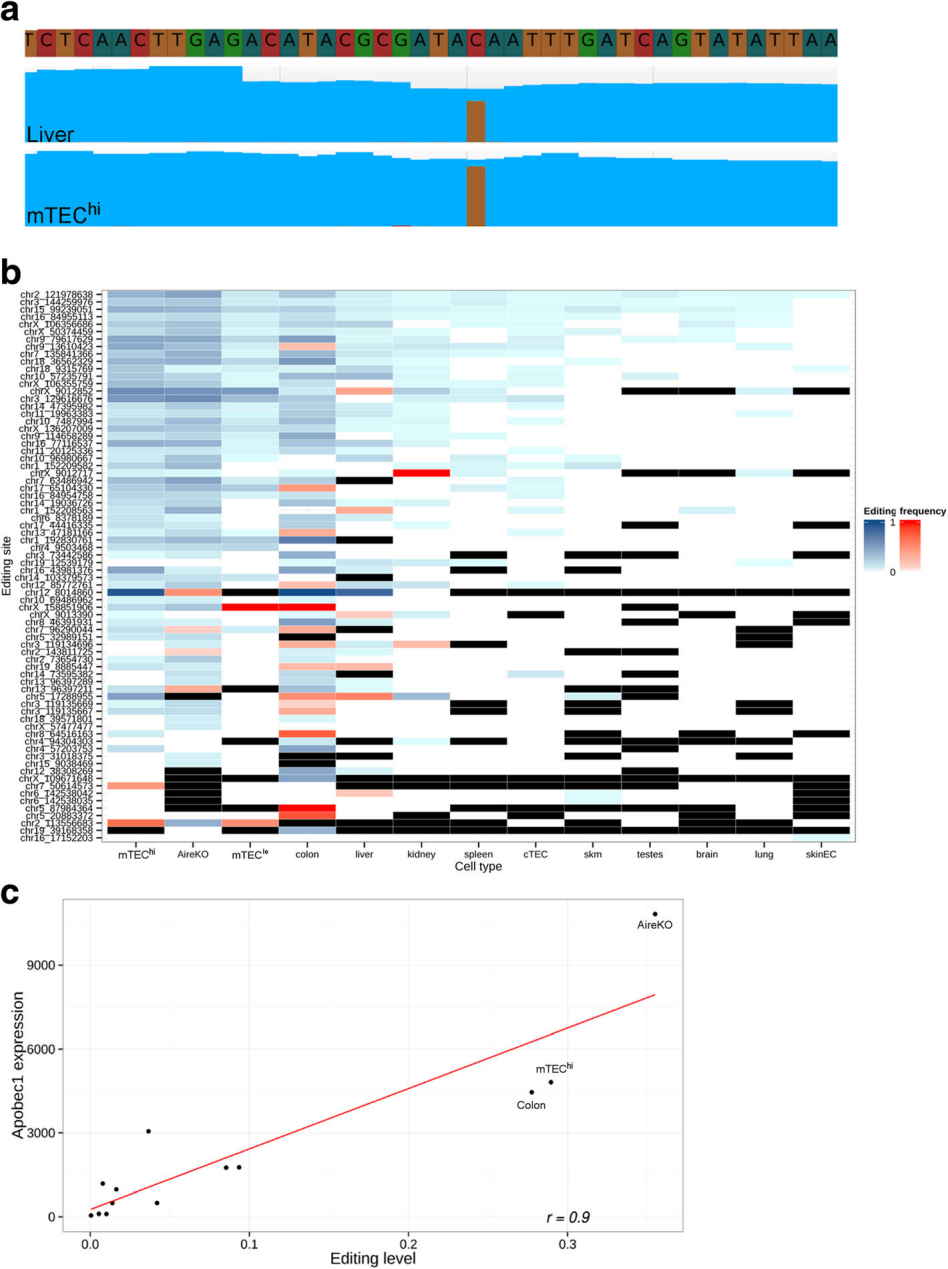
A high rate of RNA-editing sites are detected in mTEC cells (C to U). **a** C-to-U editing in ApoB gene in mTECs. Reads coverage in the ApoB C-to-U editing site (chr12:8014835–8014884), *blue* denotes the same nucleotide in the genome and in the mapped reads, *brown* denotes a T nucleotide in the reads instead of a C in the genome. **b** C-to-U editing levels in mTECs is comparable to the levels in colon. *Heatmap* of RNA-editing frequency in 77 sites originally found in the liver and colon edited in at least one of the examined cell types or tissues. *Blue rectangle* denotes editing site supported by ten reads or more (edited and non-edited reads), *red rectangle* denotes editing site supported by less than ten reads, *white rectangle* denotes no edited read was found, *black rectangle* denotes no reads were mapped to this editing site. **c** High levels of Apobec1 expression in mature mTECs correlate with high C-to-U editing levels. Correlation of Apobec1 expression levels (DEseq normalized reads count [71]) and editing levels (the number of edited reads detected in all 77 sites examined out of all reads detected in these sites). AireKO, mTEC^hi^, and colon sample values are specified in the plot

Next, we examined the level of C-to-U RNA editing, which is a less prevalent type of RNA-editing mechanism. The only verified non-synonymous change induced by this type of editing in mouse is ApoB in which a codon is edited to a termination codon in mouse liver and small intestine [24, 25, 50, 51]. Since ApoB is a bona fide TRA gene, which is expressed by mTECs in an Aire-dependent level, we first analyzed whether its C-to-U editing is also detectable in mTECs. Indeed, our analysis demonstrated a comparable level of C-to-U editing of the ApoB transcript in both liver and mTECs (Fig. 5a).

Moreover, to get a more comprehensive picture, we next analyzed the level of C-to-U editing at all putative C-to-U sites that were reported thus far in mouse. Indeed, this analysis revealed that mTECs display a relatively high C-to-U RNA-editing frequency compared to other tissues (Fig. 5b). Specifically, we found that 75 % of colon-specific and liver-specific editing sites [27, 28] that were covered by reads in mature mTECs sample were also edited there (Fig. 5b). Furthermore, although Aire-deficient mTECs displayed less detectable transcripts undergoing C-to-U editing than their WT counterparts, the RNA-editing rate for those that were covered was comparable (Fig. 5b). In contrast, mTEC^lo^ or cTECs editing rates were much lower than that obtained for mature mTECs. Moreover, *Apobec1* expression levels were comparable in mTEC^hi^ cells and in the colon and were significantly correlated to the corresponding C-to-U editing levels (Fig. 5c, Additional file 1: Table S3). Intriguingly, *Apobec1* expression levels and C-to-U editing levels were very high in AireKO sample (Fig. 5c), further supporting that this mechanism is not regulated by Aire.

## Discussion

The development of T lymphocytes in the thymus is governed by two separate lineages of thymic epithelial cells, the cortical and the medullary, which differ in their anatomical localization and their functional properties [52]. While cTECs control the early steps of T cell development, including T lineage commitment, expansion, and subsequent positive selection of double positive (DP) thymocytes [53], mTECs play a primary role in the later stages, including negative selection of self-reactive T cells and generation of thymic regulatory T cells [54, 55]. Crucial to the key role of mTECs in the establishment of immunological tolerance to self is their unique capacity to promiscuously express an exceptionally large number of tissue-restricted antigen genes. Although the physiological significance of this phenomenon is well-established, the question to what extent does their transcriptome complexity, which is further expanded by RNA editing and AS, differs from other tissues in the body has not yet been addressed in a comprehensive manner. Therefore, to better address this important question, we sought to determine the extent and diversity of promiscuous gene expression, AS, and RNA editing in this unique cell population in comparison to other cell types and tissues.

**Fig. 6:**
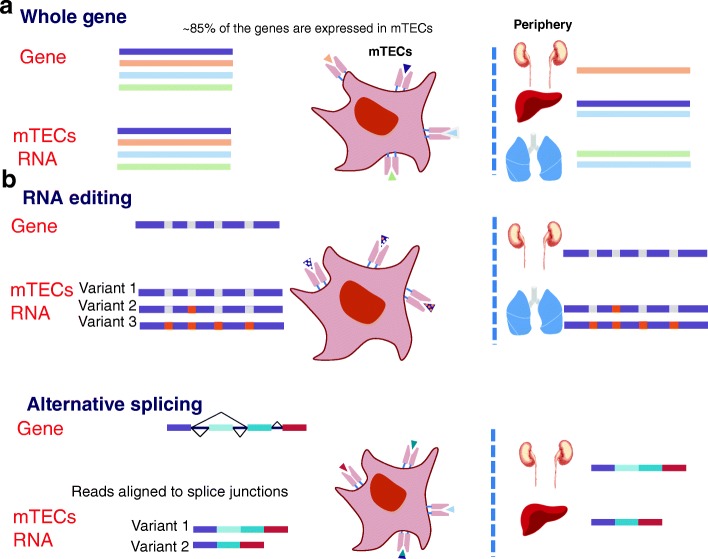
Self-representation in mTECs is expanded by extensive RNA processing. **a** *Schematic representation* of the comprehensive gene expression in the mTECs. These genes include many of the various tissue specific genes. All are further translated into antigens which are represented to T cells. **b** In this study, we further show very high levels of RNA editing and AS in mTECs. These RNA processes also result in tissue-specific antigens represented in mTECs

Indeed, comprehensive RNA-seq analysis validated that in contrast to all other analyzed cell types and tissues, mature mTECs are capable of expressing the vast majority of the protein-coding genome (Fig. 6a). Specifically, while most of the tissues express 12,000–14,000 genes (i.e. 60–65 % of the coding genome), the mTEC^hi^ population expresses nearly 18,000 genes, which represents ~85 % of the coding genome. Interestingly, the mTEC^lo^ population also demonstrated the capacity to express most of the genome, albeit at very low levels. Such a low-level promiscuous gene expression may either suggest that the transcription of most of the genes is initiated (at low levels) already at the stage of immature mTECs or that the analyzed mTEC^lo^ population also contains terminally differentiated mTECs, which have downregulated their MHC-II molecules, but still express residual levels of TRA transcripts. Given that Aire becomes expressed only at the mTEC^hi^ stage and that the mTEC^lo^ population was indeed found to contain a fraction of such post-Aire mTECs [56–58], the latter possibility seems to be more likely. In contrast, the number of genes expressed by cTECs was found to be similar to that of other analyzed tissues, suggesting that the phenomenon of promiscuous gene expression is exclusive only to terminally differentiated mTECs.

Moreover, even in the absence of Aire, the extent of genome expression in the mTEC^hi^ population is higher (~15 k) than in most of the other analyzed tissues (~12–14 k), except for the testis (~15 k). These results validate some of the previous observations suggesting that a large fraction of TRA genes (i.e. 30–40 %) is expressed by mTECs in an Aire-independent manner [59]. The molecular mechanisms controlling the expression of this Aire-independent fraction, however, remain elusive.

Importantly, our analysis also reveals that most of the analyzed peripheral tissues are, to a large extent (~60–100 %), mirrored in the mTEC^hi^ population. The only exception is the brain and testes, which display relatively little tissue-specific overlap with mTECs (55 % and 31 %, respectively). Interestingly, both tissues are considered to be immunologically privileged sites, which are not under conventional immunological surveillance. Thus, our data suggest that the stringency of central tolerance mechanisms to these two immunoprivileged sites is lower than to other peripheral tissues with conventional immunological surveillance. Consequently, more brain-reactive and testes-reactive T cell clones may escape from the thymus to the periphery, where they must be kept in check by other tolerance mechanisms, such as antigen sequestering. A good example is myelin oligodendrocyte glycoprotein (MOG), which is not expressed by mTECs, yet it is tolerated by the immune system, due to its sequestering in the brain. However, when MOG peptides become exposed (e.g. after tissue damage or immunization), the peripheral MOG-specific T cells, that escaped central tolerance, may become activated and mount an autoimmune attack on the brain [60]. Similarly, exposure of testis-specific antigens, e.g. after testicular injury, can induce a breakdown of self-tolerance mechanisms and result in autoimmune orchitis [61].

Furthermore, our analysis also revealed that mTECs are characterized by a high rate of alternatively spliced variants in comparison to other cell types and tissues (Fig. 6b). This is in line with a previously published study demonstrating high entropy of AS products in mTECs [42]. Isoform entropy is a global disorder measurement that typically increases because of widespread flattened (unbiased) isoform expression profiles [62]. Here, based on our analysis, we further expand this observation by showing that the high entropy resulted from a high percentage of alternatively spliced genes, a high number of splice variants for each gene, and a flattened isoform expression profile in mTECs. A good example to illustrate the ability of mTECs to generate various tissue-restricted variants is the Src tyrosine kinase Fyn, which is highly expressed in two different alternative forms in the hematopoietic system and in the brain. Indeed, both isoforms were expressed at comparable levels in mTECs.

Nevertheless, our results also show that there is a considerable portion of tissue-restricted splice variants that are not expressed in mTECs. These results therefore suggest that although mTECs demonstrate a dramatically higher rate of AS than other tissues, it seems to be less robust and comprehensive than their promiscuous gene expression capacity. This observation may be partly explained by the stochastic nature of the splicing machinery which gives rise to splice variants that probably represent “noise” [63, 64]. Still, the results imply that there is a relatively high probability for splice isoforms that are being expressed in peripheral tissues and not in the thymus, which may then increase susceptibility to autoimmunity, as was suggested for the PLP gene [30].

Our data suggest that Aire, which induces expression of many tissue-restricted antigen genes, does not seem to have a direct role in increasing the variety of tissue-restricted RNA-splicing products. Recently, it has been suggested that Aire regulates the inclusion of thousands of exons [39, 42]. However, our analyses imply minor changes in AS between AireKO and WT mTECs. Specifically, we observed significant differences in the expression of testes-specific and kidney-specific splice variants between the AireKO and WT mTECs and the lower overall rate of alternatively spliced genes in the Aire-deficient sample. Nevertheless, unlike the results obtained for whole gene expression, the differences between the coverage of most TRA variants generated by AS were similar between AireKO and WT mTECs. Hence, we suggest that Aire might have an indirect effect on the AS rate (e.g. through differential expression of specific splicing factors in AireKO mTECs).

We also found, for the first time, a high RNA-editing level (A-to-I and C-to-U) in mature mTECs (Fig. 6b). Global A-to-I and C-to-U RNA-editing levels in mature mTECs were comparable to the levels in the brain and colon, respectively, which are known to have the highest RNA-editing levels to date [27, 28, 49]. The high rate of RNA editing in mature mTECs detected here suggests that at some level most of the sites that have non-synonymous effect on the resulted protein are edited and hence presented to the T cells. It should be noted that many of the A-to-I sites examined here were originally found to be edited only in the brain, thus our A-to-I analyses may include a high number of brain-specific editing sites. Our C-to-U editing results revealed, for the first time, C-to-U editing in mTECs in a bona fide Aire-dependent TRA gene—ApoB—whose expression is restricted only to the liver and small intestine of mice [24, 25, 50, 51].

A high capacity of RNA editing was observed in Aire-deficient mTECs, but not in immature mTECs. Thus, another regulator (or regulators) probably mediates high RNA processing, specifically in mature mTECs. Moreover, it is reasonable that similar A-to-I editing regulation (but not C-to-U) probably occurs in cTECs.

## Conclusions

The high transcriptome complexity and high rate of RNA-processing TRAs we found to be expressed in mature mTECs ensure lower rate of differences of antigens between mTECs and any peripheral tissue. Specifically, our results demonstrate a high level of two types of RNA editing, A-to-I and C-to-U editing, together with a high rate and diversity of AS in mTECs. Moreover, our results suggest a limited role of Aire in the regulation of this high variability. Thus, our study highlights another layer by which mTECs expand the already broad repertoire of self-representation, which is required for the establishment of self-tolerance and prevention of autoimmunity.

## Methods

### RNA-seq library preparation

The AireKO population was isolated from individual strains (wild-type B6) with a FACSAria III cell sorter (BD), RNA was extracted and sequenced as detailed before [65]. Briefly, poly-A-selected transcriptome libraries were generated with a TruSeq V3 RNA Sample Prep according to the manufacturer’s protocols (Illumina). Paired-end (2 × 100 bp) sequencing was performed with an Illumina HiSeq2000 machine.

### RNA-seq processing

We used RNA-seq data from 13 different cell or tissue types. Details and sample sources are provided in Additional file 1: Table S1. STAR aligner (version 2.3.0) [66] was used to align each RNA-seq read uniquely to the mm9 mouse genome (default parameters).

### Data analysis

The statistical analysis was done using R (the R Project for Statistical Computing (http://www.r-project.org/)).

#### Expression and leave-one-out analyses

To compare global mTEC gene expression to other tissues, we created comparable samples by selection of 10, 20, 30, 40, and 50 million random aligned reads from each sample. The number of reads aligned to UCSC known genes track was counted using featureCounts [67] (parameters: −b -f –O –p). These counts were normalized by dividing the counts per gene by the gene length.

To assess TRA gene expression by mTECS, whole sample FPKM values were compared. FPKM was calculated using RSEM [68] for each RNA-seq sample. If not mentioned otherwise, a gene was considered as expressed if its FPKM value was > 0.3. A TRA gene was defined as gene that was highly expressed (FPKM > 5) in one of the tissues examined and was either lowly (FPKM < 0.3) or not expressed in other tissues.

In order to compare TRA expression in mTECs versus other tissues, leave-one-out procedure was obtained on comparable size RNA-seq samples (30 million randomly selected aligned reads). The procedure was performed by first excluding a given i-th sample, finding genes that are expressed uniquely in each sample (and not in the other remaining tissues), and then assessing the ratio of these genes expressed in the i-th sample. A gene was considered expressed if the normalized read counts was > 0.01.

#### Alternative splicing analysis

To determine AS frequency differences between the examined tissue and cell types, we used STAR splice junctions output file. In order to obtain comparable samples, reads longer than 80 nt were trimmed and 50 million random reads were selected from each sample. For each coding gene expressed in the tissue, we inferred whether it was alternatively spliced by finding at least two splice junctions that either start or end at the same position. We also compared the number of all the junctions detected within each gene expressed both in mTECs and each of the other tissues examined. Comparison of alternatively spliced variants was also conducted using rMATs [44]. The number of significantly altered exons in which one form was expressed in the examined tissue and two in mTECs and vice versa was compared.

The TRA junction was defined as a junction that is supported by at least ten reads in a specific tissue and not in any of the other tissues examined (mTECs excluded) and is found inside a gene that was expressed in at least additional tissue. In order to assess the extent of TRAs that result from tissue-restricted AS, we calculated the ratio of TRA junctions of each of the tissues examined expressed in mTECs and AireKO samples out of the TRA junctions expressed in the tissue. The same ratio was calculated for splice junctions of known events (Cassette exons, alternative 3’ splice site, and alternative 5’ splice site) from the AltEvents track of the UCSC genome browser [46].

#### RNA-editing analysis

To evaluate the global extent of RNA editing (A-to-I) in mTECs versus other cell types, we estimated the extent of hyper-editing, the editing rate of each RNA-seq sample, and compared the number of sites significantly altered between mTECs and other cell types.

Hyper-edited sites were identified as described in [16] using the reads that did not align to the genome at first (same parameters except read length > 80 % of total number of mismatches requirement instead 60 %). For each sample, the number of unique hyper-editing sites was normalized by the number of mapped reads.

The editing rate was computed as the percentage of editing sites in which the edited variant was expressed out of all known editable sites expressed in a given tissue. We randomly sampled 30 million aligned reads from each RNA-seq for comparable sample size. RNA-editing levels were calculated using the REDItoolKnown.py script that is part of REDItools package [69]. Mouse A-to-I editing site annotations were downloaded from the RADAR database ([18], v1 including 8109 sites) and were also inferred from the hyper-editing results (total of 22,701 sites). For C-to-U editing, site annotations were taken from [27, 28]. The editing level was computed as the percentage of the edited reads out of all reads mapped to known editable sites expressed in a given tissue. We also compared the number of editing sites significantly altered between sites expressed both in mTECs and each of the other tissues examined using Fisher’s exact test with correction to multiple testing (FDR). The same methods were used in order to evaluate the editing rate in sites with non-synonymous effect on the protein. Coding and protein effect information was obtained from Annovar [70]. For this analysis we used all the reads in each sample.

*Adar1*, *Adar2*, and *Apobec1* normalized reads counts were calculated using DEseq [71]. Mapping visualization was performed using Savant genome browser [72].

## Additional file

Additional file 1: Contains supplementary figures and tables: **Figures S1–S5.** and **Tables S1–S4.** (PDF 727 kb)

## References

[1] Klein L, Kyewski B, Allen PM, Hogquist KA (2014). Positive and negative selection of the T cell repertoire: what thymocytes see (and don’t see). Nat Rev Immunol.

[2] Derbinski J, Schulte A, Kyewski B, Klein L (2001). Promiscuous gene expression in medullary thymic epithelial cells mirrors the peripheral self. Nat Immunol.

[3] Anderson MS, Venanzi ES, Klein L, Chen Z, Berzins SP, Turley SJ (2002). Projection of an immunological self shadow within the thymus by the aire protein. Science.

[4] Peterson P, Org T, Rebane A (2008). Transcriptional regulation by AIRE: molecular mechanisms of central tolerance. Nat Rev Immunol.

[5] Nagamine K, Peterson P, Scott HS, Kudoh J, Minoshima S, Heino M (1997). Positional cloning of the APECED gene. Nat Genet.

[6] Graveley BR (2001). Alternative splicing: increasing diversity in the proteomic world. Trends Genet.

[7] Rosenthal JJC (2015). The emerging role of RNA editing in plasticity. J Exp Biol.

[8] Pan Q, Shai O, Lee LJ, Frey BJ, Blencowe BJ (2008). Deep surveying of alternative splicing complexity in the human transcriptome by high-throughput sequencing. Nat Genet.

[9] Wang ET, Sandberg R, Luo S, Khrebtukova I, Zhang L, Mayr C (2008). Alternative isoform regulation in human tissue transcriptomes. Nature.

[10] Nilsen TW, Graveley BR (2010). Expansion of the eukaryotic proteome by alternative splicing. Nature.

[11] Stamm S, Ben-Ari S, Rafalska I, Tang Y, Zhang Z, Toiber D (2005). Function of alternative splicing. Gene.

[12] Kelemen O, Convertini P, Zhang Z, Wen Y, Shen M, Falaleeva M (2013). Function of alternative splicing. Gene.

[13] Kim E, Goren A, Ast G (2008). Alternative splicing: current perspectives. Bioessays.

[14] Nishikura K (2010). Functions and regulation of RNA editing by ADAR deaminases. Annu Rev Biochem.

[15] Savva YA, Rieder LE, Reenan RA (2012). The ADAR protein family. Genome Biol.

[16] Porath HT, Carmi S, Levanon EY (2014). A genome-wide map of hyper-edited RNA reveals numerous new sites. Nat Commun.

[17] Burns CM, Chu H, Rueter SM, Hutchinson LK, Canton H, Sanders-Bush E (1997). Regulation of serotonin-2C receptor G-protein coupling by RNA editing. Nature.

[18] Ramaswami G, Li JB (2014). RADAR: a rigorously annotated database of A-to-I RNA editing. Nucleic Acids Res.

[19] Danecek P, Nellåker C, McIntyre RE, Buendia-Buendia JE, Bumpstead S, Ponting CP (2012). High levels of RNA-editing site conservation amongst 15 laboratory mouse strains. Genome Biol.

[20] Bazak L, Haviv A, Barak M, Jacob-Hirsch J, Deng P, Zhang R (2014). A-to-I RNA editing occurs at over a hundred million genomic sites, located in a majority of human genes. Genome Res.

[21] O’Connell MA, Keller W (1994). Purification and properties of double-stranded RNA-specific adenosine deaminase from calf thymus. Cell Biol.

[22] Smith HC, Bennett RP, Kizilyer A, McDougall WM, Prohaska KM (2012). Functions and regulation of the APOBEC family of proteins. Semin Cell Dev Biol.

[23] Blanc V, Davidson NO (2010). APOBEC-1-mediated RNA editing. Wiley Interdiscip Rev Syst Biol Med.

[24] Powell LM, Wallis SC, Pease RJ, Edwards YH, Knott TJ, Scott J (1987). A novel form of tissue-specific RNA processing produces apolipoprotein-B48 in intestine. Cell.

[25] Chen S, Habib G, Yang C, Gu Z, Lee B, Weng S (1987). Apolipoprotein B-48 is the product of a messenger RNA with an organ-specific in-frame stop codon. Science.

[26] Sharma S, Patnaik SK, Taggart RT, Kannisto ED, Enriquez SM, Gollnick P (2015). APOBEC3A cytidine deaminase induces RNA editing in monocytes and macrophages. Nat Commun.

[27] Blanc V, Park E, Schaefer S, Miller M, Lin Y, Kennedy S (2014). Genome-wide identification and functional analysis of Apobec-1-mediated C-to-U RNA editing in mouse small intestine and liver. Genome Biol.

[28] Rosenberg BR, Hamilton CE, Mwangi MM, Dewell S, Papavasiliou FN (2011). Transcriptome-wide sequencing reveals numerous APOBEC1 mRNA-editing targets in transcript 3′ UTRs. Nat Struct Mol Biol.

[29] Kyewski B, Derbinski J (2004). Self-representation in the thymus: an extended view. Nat Rev Immunol.

[30] Anderson AC, Nicholson LB, Legge KL, Turchin V, Zaghouani H, Kuchroo VK (2000). High frequency of autoreactive myelin proteolipid protein-specific T cells in the periphery of naive mice: mechanisms of selection of the self-reactive repertoire. J Exp Med.

[31] Kyewski B, Klein L (2006). A central role for central tolerance. Annu Rev Immunol.

[32] Merkin J, Russell C, Chen P, Burge CB (2012). Evolutionary dynamics of gene and isoform regulation in Mammalian tissues. Science.

[33] St-Pierre C, Brochu S, Vanegas JR, Dumont-Lagacé M, Lemieux S, Perreault C (2013). Transcriptome sequencing of neonatal thymic epithelial cells. Sci Rep.

[34] Soumillon M, Necsulea A, Weier M, Brawand D, Zhang X, Gu H (2013). Cellular source and mechanisms of high transcriptome complexity in the mammalian testis. Cell Rep.

[35] Ramsköld D, Wang ET, Burge CB, Sandberg R (2009). An abundance of ubiquitously expressed genes revealed by tissue transcriptome sequence data. PLoS Comput Biol.

[36] Johnson MB, Kawasawa YI, Mason CE, Krsnik Z, Coppola G, Bogdanović D (2009). Functional and evolutionary insights into human brain development through global transcriptome analysis. Neuron.

[37] Schmidt EE (1996). Transcriptional promiscuity in testes. Curr Biol.

[38] Sansom SN, Shikama-Dorn N, Zhanybekova S, Nusspaumer G, Macaulay IC, Deadman ME (2014). Population and single-cell genomics reveal the Aire dependency, relief from Polycomb silencing, and distribution of self-antigen expression in thymic epithelia. Genome Res.

[39] Meredith M, Zemmour D, Mathis D, Benoist C (2015). Aire controls gene expression in the thymic epithelium with ordered stochasticity. Nat Immunol.

[40] Brennecke P, Reyes A, Pinto S, Rattay K, Nguyen M, Küchler R (2015). Single-cell transcriptome analysis reveals coordinated ectopic gene-expression patterns in medullary thymic epithelial cells. Nat Immunol.

[41] Kyewski B, Derbinski J, Gotter J, Klein L (2002). Promiscuous gene expression and central T-cell tolerance: more than meets the eye. Trends Immunol.

[42] Keane P, Ceredig R, Seoighe C (2015). Promiscuous mRNA splicing under the control of AIRE in medullary thymic epithelial cells. Bioinformatics.

[43] Grabowski PJ, Black DL (2001). Alternative RNA splicing in the nervous system. Prog Neurobiol.

[44] Shen S, Park JW, Lu Z, Lin L, Henry MD, Wu YN (2014). rMATS: Robust and flexible detection of differential alternative splicing from replicate RNA-Seq data. Proc Natl Acad Sci.

[45] Cooke MP, Perlmutter RM (1989). Expression of a novel form of the fyn proto-oncogene in hematopoietic cells. New Biol.

[46] Karolchik D, Baertsch R, Diekhans M, Furey TS, Hinrichs A, Lu YT (2003). The UCSC Genome Browser Database. Nucleic Acids Res.

[47] Farajollahi S, Maas S (2010). Molecular diversity through RNA editing: a balancing act. Trends Genet.

[48] Conticello SG (2008). The AID/APOBEC family of nucleic acid mutators. Genome Biol.

[49] Paul MS, Bass BL (1998). Inosine exists in mRNA at tissue-specific levels and is most abundant in brain mRNA. EMBO J.

[50] Young SG (1990). Recent progress in understanding apolipoprotein B. Circulation.

[51] Higuchi K, Kitagawa K, Kogishi K, Takeda T (1992). Developmental and age-related changes in apolipoprotein B mRNA editing in mice. J Lipid Res.

[52] Anderson G, Takahama Y (2012). Thymic epithelial cells: working class heroes for T cell development and repertoire selection. Trends Immunol.

[53] Stritesky GL, Jameson SC, Hogquist KA (2012). Selection of self-reactive T cells in the thymus. Annu Rev Immunol.

[54] Aschenbrenner K, D’Cruz LM, Vollmann EH, Hinterberger M, Emmerich J, Swee LK (2007). Selection of Foxp3+ regulatory T cells specific for self antigen expressed and presented by Aire + medullary thymic epithelial cells. Nat Immunol.

[55] Cowan JE, Parnell SM, Nakamura K, Caamano JH, Lane PJL, Jenkinson EJ (2013). The thymic medulla is required for Foxp3+ regulatory but not conventional CD4+ thymocyte development. J Exp Med.

[56] Wang ET, Cody NAL, Jog S, Biancolella M, Wang TT, Treacy DJ (2012). Transcriptome-wide regulation of pre-mRNA splicing and mRNA localization by muscleblind proteins. Cell.

[57] Metzger TC, Khan IS, Gardner JM, Mouchess ML, Johannes KP, Krawisz AK (2013). Lineage tracing and cell ablation identify a post-Aire-expressing thymic epithelial cell population. Cell Rep.

[58] Lkhagvasuren E, Sakata M, Ohigashi I, Takahama Y (2013). Lymphotoxin β receptor regulates the development of CCL21-expressing subset of postnatal medullary thymic epithelial cells. J Immunol.

[59] Derbinski J, Gäbler J, Brors B, Tierling S, Jonnakuty S, Hergenhahn M (2005). Promiscuous gene expression in thymic epithelial cells is regulated at multiple levels. J Exp Med.

[60] Amor S, Groome N, Linington C, Morris MM, Dornmair K, Gardinier MV (1994). Identification of epitopes of myelin oligodendrocyte glycoprotein for the induction of experimental allergic encephalomyelitis in SJL and Biozzi AB/H mice. J Immunol.

[61] Sakamoto Y, Matsumoto T, Mizunoe Y, Haraoka M, Sakumoto M, Kumazawa J (1995). Testicular injury induces cell-mediated autoimmune response to testis. J Urol.

[62] Ritchie W, Granjeaud S, Puthier D, Gautheret D (2008). Entropy measures quantify global splicing disorders in cancer. PLoS Comput Biol.

[63] Pickrell JK, Pai AA, Gilad Y, Pritchard JK (2010). Noisy splicing drives mRNA isoform diversity in human cells. PLoS Genet.

[64] Melamud E, Moult J (2009). Stochastic noise in splicing machinery. Nucleic Acids Res.

[65] Chuprin A, Avin A, Goldfarb Y, Herzig Y, Levi B, Jacob A (2015). The deacetylase Sirt1 is an essential regulator of Aire-mediated induction of central immunological tolerance. Nat Immunol.

[66] Dobin A, Davis CA, Schlesinger F, Drenkow J, Zaleski C, Jha S (2013). STAR: ultrafast universal RNA-seq aligner. Bioinformatics.

[67] Liao Y, Smyth GK, Shi W (2013). featureCounts: an efficient general purpose program for assigning sequence reads to genomic features. Bioinformatics.

[68] Li B, Dewey CN (2011). RSEM: accurate transcript quantification from RNA-Seq data with or without a reference genome. BMC Bioinformatics.

[69] Picardi E, Pesole G (2013). REDItools: high-throughput RNA editing detection made easy. Bioinformatics.

[70] Wang K, Li M, Hakonarson H (2010). ANNOVAR: functional annotation of genetic variants from high-throughput sequencing data. Nucleic Acids Res.

[71] Anders S, Huber W (2010). DESeq: Differential expression analysis for sequence count data. Genome Biol.

[72] Fiume M, Williams V, Brook A, Brudno M (2010). Savant: genome browser for high-throughput sequencing data. Bioinformatics.

