# Supplementary material for: Extensive RNA editing and splicing increase immune self-representation diversity in medullary thymic epithelial cells
Source: Genome Biol. 2016 Oct 24;17:219. doi: 10.1186/s13059-016-1079-9 (PMC5078920; doi:10.1186/s13059-016-1079-9)
Supplement: Additional file 1: — Contains supplementary figures and tables: Figures S1–S5. and Tables S1–S4. (PDF 727 kb) [file 13059_2016_1079_MOESM1_ESM.pdf]

Extensive RNA editing and splicing increase immune self-representation diversity in medullary thymic epithelial cells

Supplementary Information

A

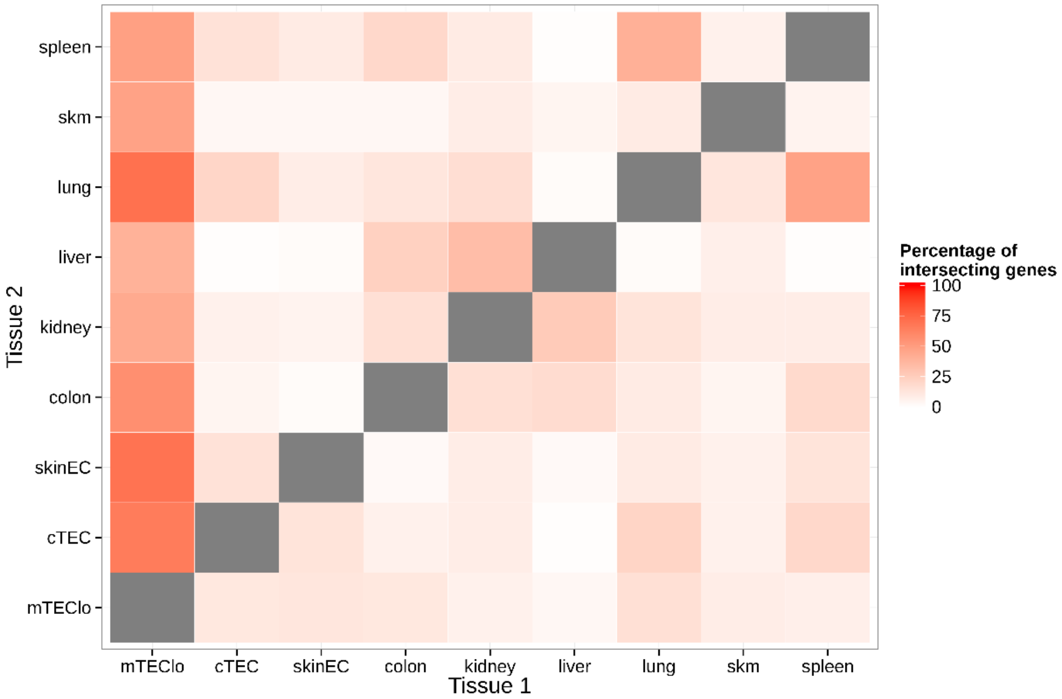

B

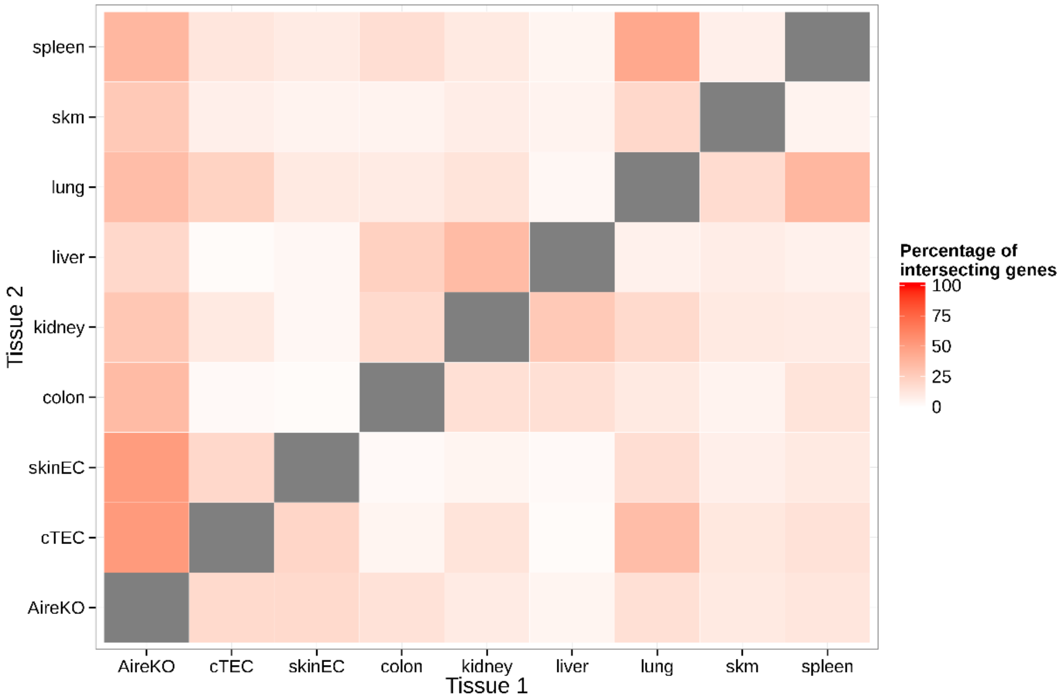

Figure S1. mTEC cells express higher number of tissue specific genes compare to other tissues. Leave-one-out analysis. The percentage of the coding genes uniquely expressed in both tissue 1 and tissue 2 (and not in other tissue examined) out of the genes expressed only in tissue 1 (and not in other tissue except tissue 2) are presented. The analysis was performed on genes expressed the indicated tissues and A. mTEClo cells. B. Aire KO cells.

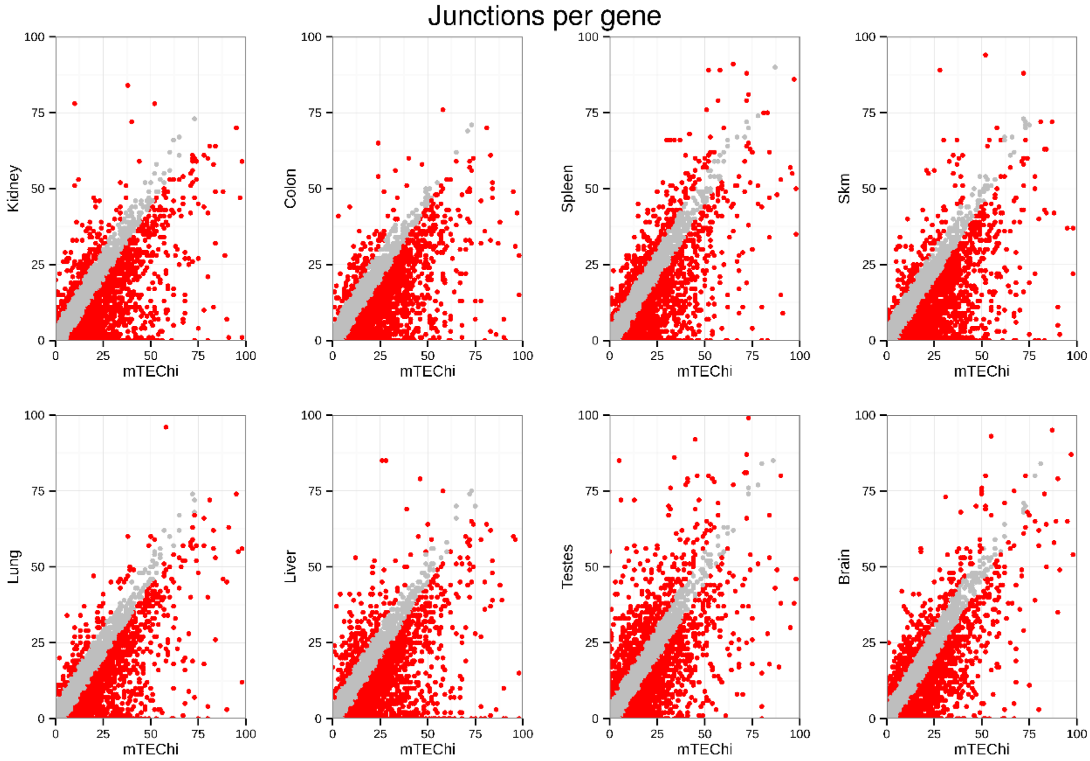

Figure S2. Correlation of splice junctions number detected for each gene expressed in both mTEChi and each sample examined. Using only genes with expression level of less than 2 FPKM. Red dots denote difference of more than 5 splice junctions per gene, grey dots denote 5 and less splice junctions difference per gene.

A

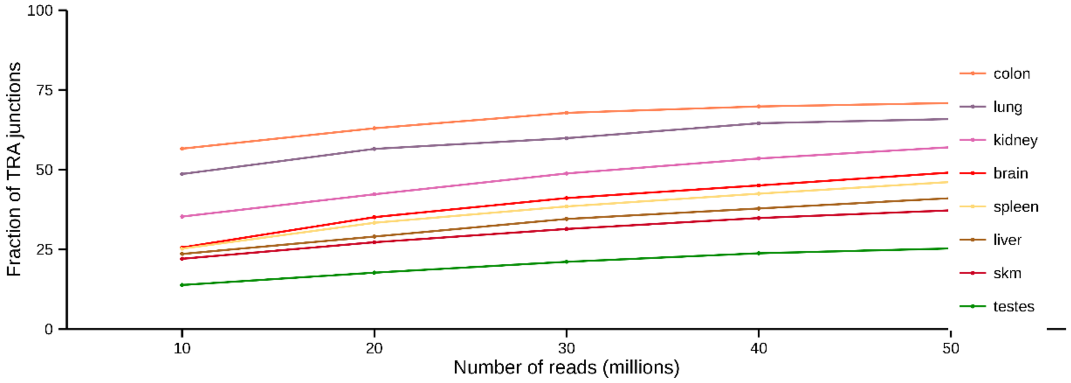

B

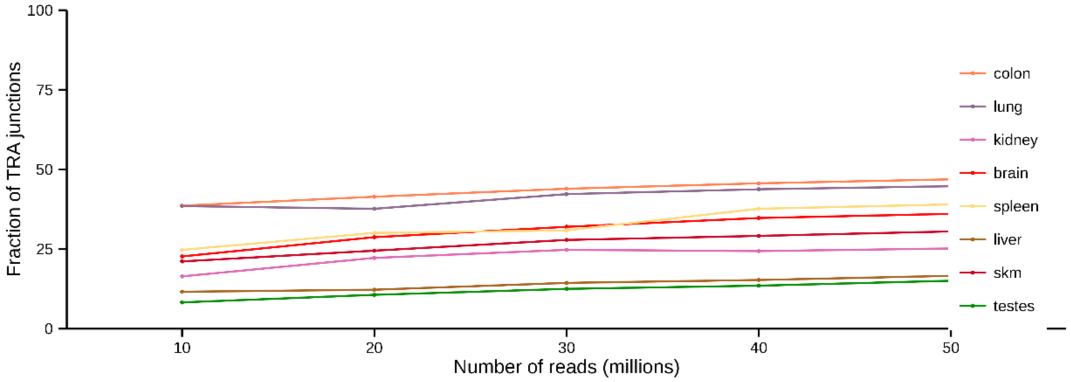

Figure S3. The ratio of TRA junctions covered by mTECs is almost not coverage-dependent. The ratio of TRA junctions of each tissue expressed in mTECs on the basis of 10–60 million randomly selected aligned RNA-seq reads for A. mTEC^{hi} sample. B. AireKO sample.

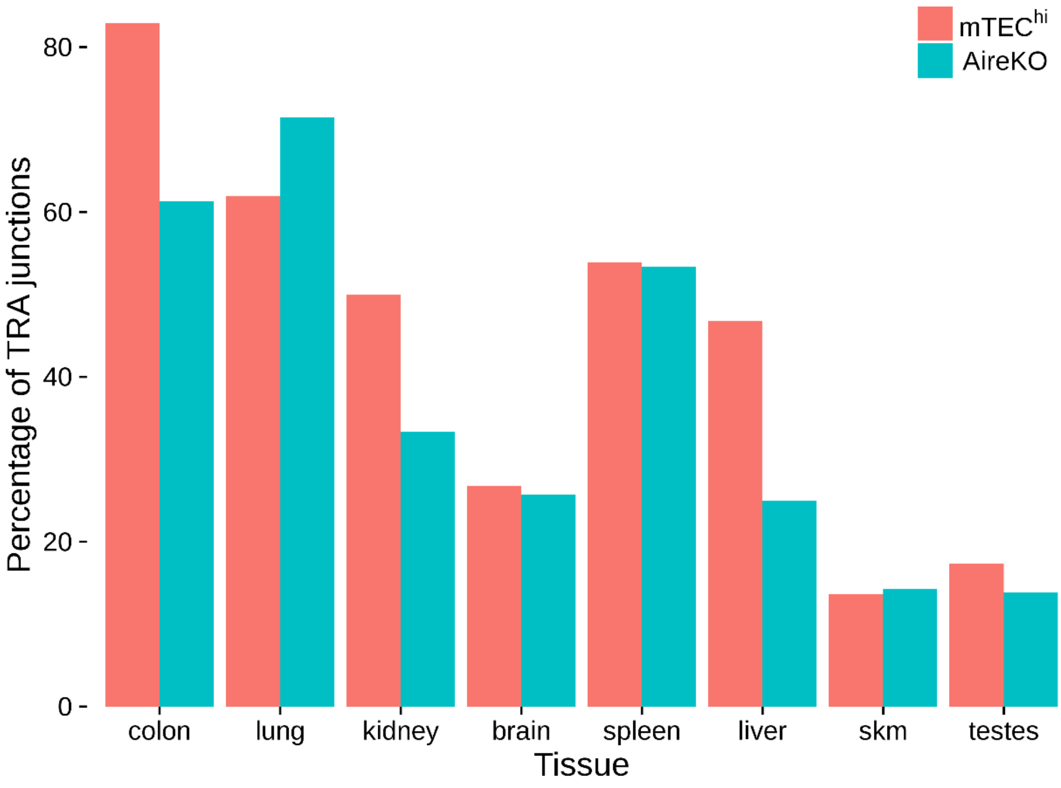

Figure S4. Fraction of TRA junctions expressed in mature mTECs and Aire-KO sample using only junctions annotated in the UCSC track "Alt events". Lower fraction of brain and skeletal muscle specific junctions were expressed by mTECs. These differences were probably originated from biases in the annotation of these tissues since the rates of mTECs TRA junctions expressed in other tissues remained similar.

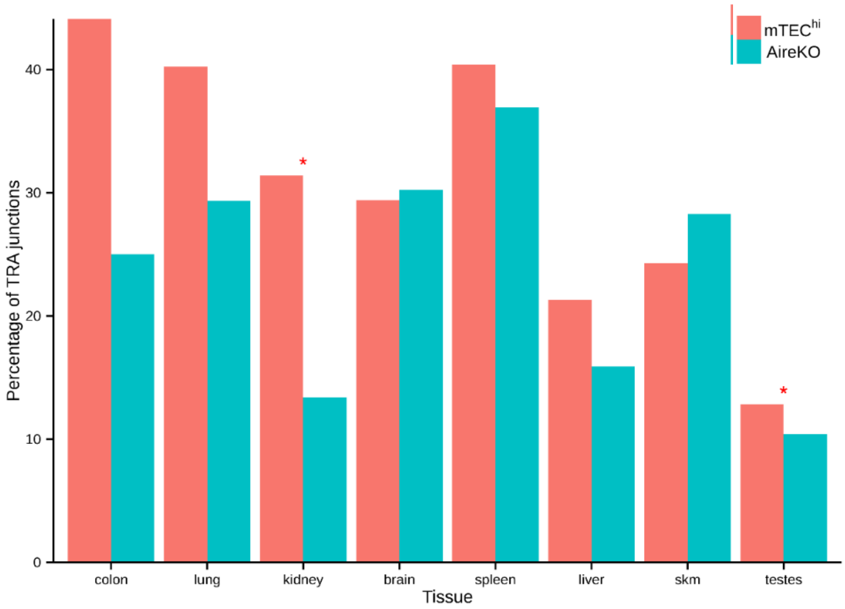

Figure S5. Fraction of TRA junctions expressed in mature mTECs and Aire-KO sample using only highly expressed genes (FPKM>2). Asterisk denotes a statistically significant change between $mTEC^{hi}$ and AireKO sample (p-value <= 0.05).

Table S1. RNA-seq samples details used in this study

Sample	Source	Number of reads	Read length	Number of aligned reads
mTEChi	[1]	150,909,756	101x2	117,686,912
mTEClo	[1]	100,370,963	101x2	69,897,326
AireKO	This study	144,478,963	101x2	120,871,736
colon	[2]	87,447,334	80x2	71,999,792
Kidney	[2]	118,885,190	80,75	102,489,254
Brain	[2]	118,824,353	80x2	108,277,535
Testes	[2]	116,525,147	80x2	106,672,396
Spleen	[2]	114,814,142	80x2	102,166,283
Skeletal muscle	[2]	117,171,737	80x2	102,286,096
Liver	[2]	134,045,721	80x2	113,343,055
Lung	[2]	62,362,901	80x2	54,579,729
cTEC	[3]	57,384,178	100x2	31,050,981
Skin epithelial cell	[3]	42,902,367	100x2	31,178,417

Table S2. Number of variants uniquely expressed in mTECs vs. number of variants expressed in the corresponding tissue and not in mTECs

Tissue	Number of variants expressed in mTECs and not in the tissue	Number of variants expressed in the tissue and not in mTECs
Brain	1168	1289
Colon	379	114
Liver	693	345
Lung	494	285
Skeletal muscle	999	947
Spleen	728	501
Testes	2073	1528

Table S3. Adar and Apobce1 expression in different tissues

Gene	$mTEC^{hi}$	$mTEC^{lo}$	AireKO	cTEC	skinEC	Brain	Testes	Colon	spleen	liver	skm	lung	kidney
Adar	4643	11566	6750	11226	3147	12217	1371	6317	11166	5044	818	4169	3197
Adar2	602	514	659	2353	1281	18546	2135	158	481	2710	1707	3784	1197
Apobec1	4814	1763	10831	974	48	108	103	4457	3056	1753	496	1181	497

*Values are given as normalized count reads (Deseq)

Table S4. Number of editing sites edited at significantly higher levels in mTECs vs. Number of editing sites edited at significantly higher levels in the corresponding tissue

Tissue	Number of editing sites edited at significantly higher levels in the tissue	Number of editing sites edited at significantly higher levels in mTECs
Colon	4	38
Kidney	2	118
Brain	70	115
Testes	1	111
Skeletal muscle	2	156
Spleen	13	43
Liver	5	57
Lung	6	28
$mTEC^{lo}$	39	7
cTEC	5	28
skinEC	5	332
AireKO	2	54

References

1. Chuprin A, Avin A, Goldfarb Y, Herzig Y, Levi B, Jacob A, Sela A, Katz S, Grossman M, Guyon C, Rathaus M, Cohen HY, Sagi I, Giraud M, McBurney MW, Husebye ES, Abramson J: The deacetylase Sirt1 is an essential regulator of Aire-mediated induction of central immunological tolerance. Nat Immunol 2015, 16:737–45.
2. Merkin J, Russell C, Chen P, Burge CB: Evolutionary dynamics of gene and isoform regulation in Mammalian tissues. Science 2012, 338:1593–9.
3. St-Pierre C, Brochu S, Vanegas JR, Dumont-Lagacé M, Lemieux S, Perreault C: Transcriptome sequencing of neonatal thymic epithelial cells. Sci Rep 2013, 3:1860.
